# Whole Genome Sequencing Revealed Mutations in Two Independent Genes as the Underlying Cause of Retinal Degeneration in an Ashkenazi Jewish Pedigree

**DOI:** 10.3390/genes8090210

**Published:** 2017-08-24

**Authors:** Kevin Gustafson, Jacque L. Duncan, Pooja Biswas, Angel Soto-Hermida, Hiroko Matsui, David Jakubosky, John Suk, Amalio Telenti, Kelly A. Frazer, Radha Ayyagari

**Affiliations:** 1 Ophthalmology, University of California, San Francisco, San Francisco, CA 94143-0730, USA; kevingustafson11@gmail.com; 2 REVA University, Bengaluru, Karnataka 560034, India; pobiswas@ucsd.edu; 3 Shiley Eye Institute, University of California, San Diego, La Jolla, CA 92093-0946, USA; ashermida@ucsd.edu (A.S.-H.); jjsuk@ucsd.edu (J.S.); rayyagari@ucsd.edu (R.A.); 4 Biomedical Sciences Graduate Program, University of California, San Diego, La Jolla, CA 92093, USA; hmatsui@ucsd.edu (H.M.); djakubos@ucsd.edu (D.J.); kafrazer@ucsd.edu (K.A.F.); 5 Human Longevity, Inc., San Diego, CA 92121, USA; atelenti@humanlongevity.com; 6 Department of Pediatrics, Rady Children’s Hospital, Division of Genome Information Sciences, San Diego, CA 92093, USA

**Keywords:** retina, genetics, retinitis pigmentosa

## Abstract

Retinitis pigmentosa (RP) causes progressive photoreceptor loss resulting from mutations in over 80 genes. This study identified the genetic cause of RP in three members of a non-consanguineous pedigree. Detailed ophthalmic evaluation was performed in the three affected family members. Whole exome sequencing (WES) and whole genome sequencing (WGS) were performed in the three affected and the two unaffected family members and variants were filtered to detect rare, potentially deleterious variants segregating with disease. WES and WGS did not identify potentially pathogenic variants shared by all three affected members. However, WES identified a previously reported homozygous nonsense mutation in *KIZ* (c.226C>T, p.Arg76*) in two affected sisters, but not in their affected second cousin. WGS revealed a novel 1.135 kb homozygous deletion in a retina transcript of *C21orf2* and a novel 30.651 kb heterozygous deletion in *CACNA2D4* in the affected second cousin. The sisters with the *KIZ* mutation carried no copies of the *C21orf2* or *CACNA2D4* deletions, while the second cousin with the *C21orf2* and *CACNA2D4* deletions carried no copies of the *KIZ* mutation. This study identified two independent, homozygous mutations in genes previously reported in autosomal recessive RP in a non-consanguineous family, and demonstrated the value of WGS when WES fails to identify likely disease-causing mutations.

## 1. Introduction

Retinal degenerations (RD) are hereditary diseases that cause progressive loss of vision as a result of mutations in genes critical to photoreceptor and retinal pigment epithelium (RPE) function and survival [1]. Retinitis pigmentosa (RP) is one of the most common forms of RD, with a prevalence of approximately 1/4000 and affecting an estimated 100,000 people in the United States [2]. It is characterized by progressive dysfunction and degeneration of rod photoreceptors, followed by death of cone photoreceptors and RPE cells. The symptoms begin as night blindness and progress to loss of peripheral vision and eventually central vision years later. RP is genetically transmitted and inheritance patterns include autosomal dominant, autosomal recessive, X-linked, and mitochondrial. To date, at least 82 genes have been identified that cause RP [3]. In addition to the allelic heterogeneity, phenotypic heterogeneity further complicates the characterization of RP patients, as patients with the same genetic mutation can present with varying severity of disease and effects on different retinal locations. However, new genome analysis approaches offer opportunities to test multiple genes, whole exomes, and even whole genomes in families. These modalities provide valuable insight into the underlying genetic cause of RP in individuals and families and can improve understanding of the disease and its progression.

The present study characterized the phenotype and identified the genetic basis of RP in two affected siblings and an affected second cousin in a non-consanguineous, four-generation pedigree. This study revealed the utility of comprehensive genetic analysis to identify multiple genes that may be responsible for RD in the same family.

## 2. Materials and Methods

Approval from the institutional review board ethics committee at University of California, San Francisco (UCSF) was obtained before undertaking the research (project number 071869). Retrospective review of ophthalmic examination notes was performed in three affected family members (II:2, II:4, and II:6). Information about eye examination, including best corrected visual acuity (BCVA) and dilated fundus examination, kinetic perimetry, and spectral-domain optical coherence tomography (SD-OCT) scans through the fovea were obtained in all three affected patients (Spectralis HRA+OCT, Heidelberg Engineering, Heidelberg, Germany and Cirrus HD OCT, Carl Zeiss Meditech Inc., Dublin, CA, USA). Full-field electroretinogram (ERG) testing was performed according to International Society for Clinical Electrophysiology of Vision (ISCEV) standards [4] in the two sisters (II:2 and II:4) but was not performed in the second cousin (II:6).

Whole blood samples were collected from three affected (II:2, II:4 and II:6) and two unaffected individuals (I:6 and II:3). DNA isolation was done using Puregene Blood Kit and protocol (Qiagen, Germantown, MD, USA). Whole exomes of three affected (II:2, II:4 and II:6) and to unaffected family members (I:6, II:3) were sequenced using Agilent Sure Select V1 probes (Agilent Technologies, Inc., Santa Clara, CA, USA) and Illumina HiSeq 2500 (Illumina, San Diego, CA, USA). Whole genomes of family members II:2, II:3, II:4, II:6, and I:6 were sequenced at more than 30X depth using Illumina HiSeq X (Illumina,). Sequence reads were aligned using Burrows-Wheeler Aligner (BWA) [5] against human genome 19 (hg19) with decoy sequences [6] with the default parameters, then duplicate reads were marked using Biobambam2 [7]. The aligned reads were sorted by the genomic coordinate using Sambamba processor [8] and stored in BAM format files. Single nucleotide polymorphism (SNPs), short insertion-deletion (INDELs) and single nucleotide variant (SNVs) calling was performed using Genome Analysis Tool Kit (GATK) [9]. Copy number variations (CNVs) were called using Genome STRiP and SpeedSeq software [10]. Variants were filtered to detect rare potentially deleterious variants segregating with disease. Rare deleterious SNVs were extracted based on the frequency of alleles in 1000 genome project [6] and ExAC database [11], and the deleteriousness was determined by three annotation tools: SnpEff v4.11 [12], Polyphen v2.2.2 [13], and CADD v1.3 [14]. Coding CNVs were extracted by taking overlap between all the CNVs and exons on the human genome. Segregation of substitution mutations and small deletions were tested by dideoxy sequencing [15,16]. Large deletions were validated by quantitative polymerase chain reaction (qPCR) analysis [17]. Validation of deletions was performed by quantitative real-time PCR (qRT-PCR) analysis of genomic DNA as described in Bujakowska et al. 2017 [17]. In brief, primers specific to exons 19, 21, and 26 of *CACNA2D4* and two reference genes *ZNF80* and *GPR15* were designed (Supplementary Table S1) for qRT-PCR [17]. Amplification reaction was performed for each sample separately in triplicates in a reaction mix containing 10 μL of iQ SYBR Green Supermix (Bio-Rad Laboratories, Hercules, CA, USA), 500 nM each of forward and reverse primers, and 10 ng of template DNA in a total volume of 20 μL per reaction. Each set of reactions included the analysis of calibration control human genomic DNA (Roche Applied Science, Penzberg, Germany) and two reference genes *ZNF80* and *GPR15.* Melting curve was generated at the end of each amplification cycle. Calculation of the gene copy number was carried out using 2^−ΔΔCT^ method and normalizing to two references genes *ZNF80* and *GPR15*. Standard curves were performed for each tested gene position to ensure that the amplification efficiencies of target and reference genes were similar. The standard deviation reflecting normalizing to each of the reference genes was calculated and presented as the error bars. The difference in the qRT-PCR values were calculated by performing t-test.

## 3. Results

### 3.1. Pedigree

**Figure 1:**
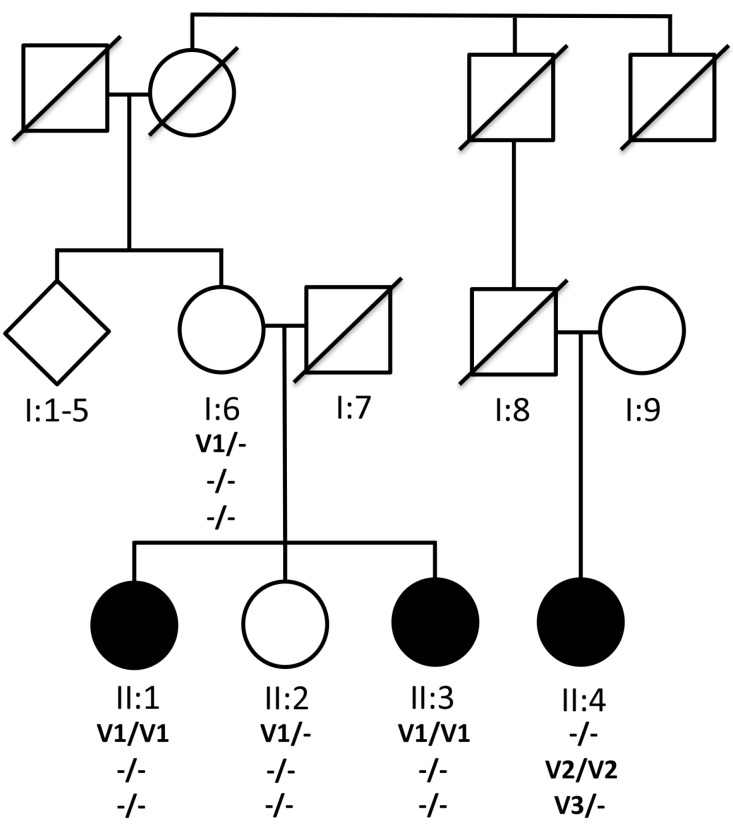
Pedigree RF.L.11.10 and segregation of mutations in *KIZ* and *C21orf2* with recessive RD. I:1–5 represents elder siblings (three unaffected males and two unaffected females) of I:6. (-) Indicates presence of wild type allele where as V1, V2 and V3 indicate the mutant alleles. The homozygous nonsense mutation p.Arg76* in *KIZ* (V1) segregated with disease in one branch of the family with affected members II:2 and II:4. A 1.1Kb homozygous deletion V2 (Chr21: 45,755,728–45,756,862) in *C21orf2* gene was observed in II:6 from a different branch of the pedigree RF.L.11.10. An additional 30Kb heterozygous deletion V3 (Chr12: 1,949,399–1,980,050) in *CACNA2D4* gene was also observed in the affected member II:6.

Two sisters (II:2 and II:4) and one female second cousin (II:6) were of Ashkenazi Jewish descent and there was no known consanguinity in the family (Figure 1). Clinical information is in Table 1.

### 3.2. Clinical Information

#### 3.2.1 Patient II:2

**Figure 2:**
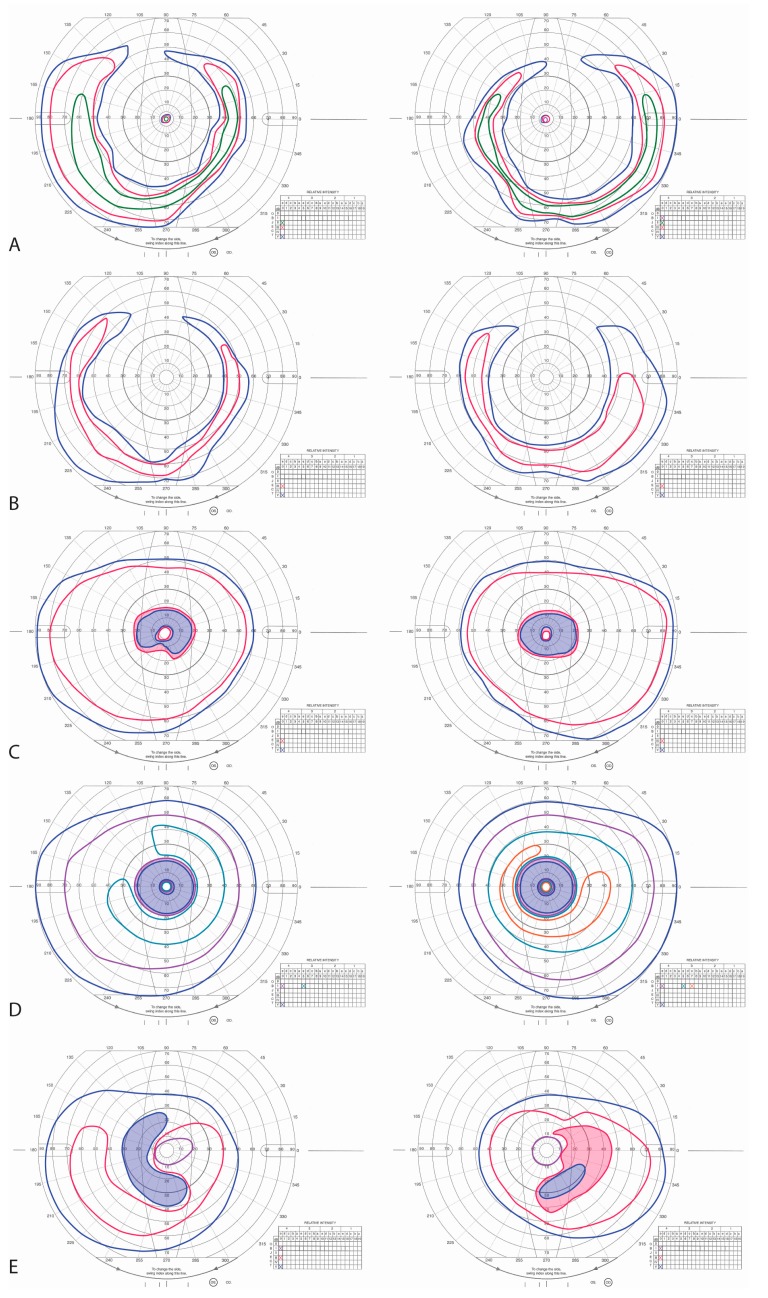
Kinetic Perimetry. (**A**) Goldmann kinetic perimetry of II:2 at age 52 shows extensive mid-peripheral ring scotomas with preserved central islands; (**B**) Visual fields at age 60 show large central scotomas without central islands; (**C**,**D**) Goldmann kinetic perimetry of II:4 at age 47 and 51, respectively, shows progressive expansion of mid-peripheral ring scotomas and preserved central islands; (**E**) Goldmann kinetic perimetry of II:6 at age 56 shows mid-peripheral ring scotomas with preserved central islands. Left panels show left visual fields while right panels show right visual fields. Shaded regions indicate scotomas.

The older sister (II:2) was a 60-year-old woman with longstanding decreased night vision. She first presented with nyctalopia at the age of 26. At that time, visual fields showed mid-peripheral visual field loss and dilated fundus exam showed bilateral epiretinal membranes with minimal pigmentary change and full-field ERG showed rod-greater-than-cone dysfunction, and an audiogram was normal. Visual fields (Figure 2A) are shown at age 52. Past medical history was significant for mild hearing loss in the lower and mid-frequency tones beginning at age 52; an audiogram at age 55 revealed hearing loss in the lower and mid-frequency tones (500–2000 Hz). Examination at age 60 revealed BCVA of 20/800 in both eyes; intraocular pressures and anterior segment examination were normal with the exception of mild nuclear sclerotic cataract in each eye. Kinetic visual fields showed large central scotomas with peripheral islands in each eye (Figure 2B).

**Figure 3:**
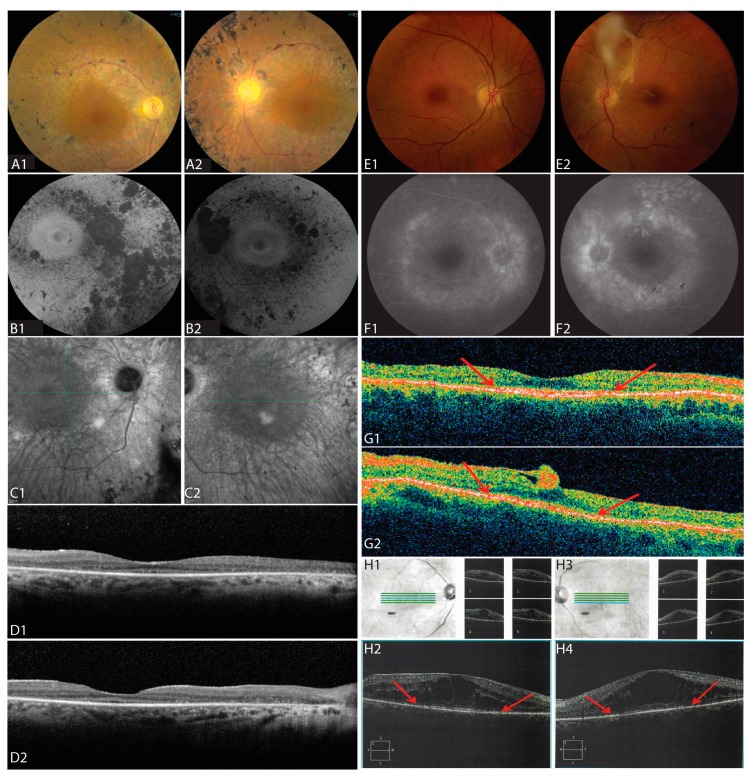
Retinal Imaging. (**A**–**D**) Fundus imaging of the right (**A1**–**D1**) and left (**A2**–**D2**) eye of II:2 at age 60. (**A1**,**A2**) Color fundus photos show disc pallor, retinal vascular attenuation and bone spicule pigmentary change along the arcades. (**B1**,**B2**) Fundus autofluorescence shows a ring of increased autofluorescence in the macula and nummular loss of autofluorescence along the arcades in both eyes. (**C1**,**C2**) Infrared fundus images show retinal pigment epithelium (RPE) loss along the arcades, vascular attenuation, and bone spicules in both eyes. (**D1**,**D2**) Macular horizontal spectral domain optical coherence tomography (SD-OCT) scans show loss of outer retinal layers throughout the macula with preserved outer nuclear layer (ONL), external limiting membrane (ELM), and inner segments (IS) at the fovea in both eyes. (**E**–**G**) Fundus imaging of the right (**E1**–**G1**) and left (**E2**–**G2**) eye of II:4 at age 47. (**E1**,**E2**) Color fundus photos of the right eye show pigment mottling along the arcades (**E1**), and photos of the left eye shows a dense epiretinal membrane with overlying vitreous opacity and pigmentary changes along the inferotemporal arcade (**E2**). (**F1**,**F2**) Fluorescein angiography shows staining of the optic nerve and areas of RPE atrophy in late frames. (**G1**,**G2**) SD-OCT scans show a preserved ONL, ELM, and inner segment/outer segment (IS/OS) junction band at the fovea in both eyes; the edges of the IS/OS junction band are demarcated by red arrows. (**H1**–**H4**) Infrared fundus images and SD-OCT B-scans of the right (**H1**,**H2**) and left (**H3**,**H4**) of patient II:6 at age 58. Blue lines indicate location of SD-OCT B-scans (bottom) and green lines show locations of smaller scans showing cystoid macular edema with large foveal cysts and IS/OS junction band loss; red arrows indicate the edges of the IS/OS junction band.

A dilated fundus exam showed mild disc pallor, cellophane epiretinal membranes, and bone spicules in both eyes (Figure 3A). There was no macular edema. Fundus autofluorescence revealed a ring of increased autofluorescence in the macula and nummular loss of autofluorescence along the arcades (Figure 3B). Infrared fundus images showed vascular attenuation and bone spicules in both eyes (Figure 3C). Macular horizontal SD-OCT scans showed loss of outer retinal layers throughout the macula, but preserved outer nuclear layer (ONL), external limiting membrane (ELM), and inner segments (IS) at the fovea in both eyes. Despite her poor central vision due to the loss of the outer segments, cone cell bodies and IS were present at the fovea in each eye (Figure 3D).

#### 3.2.2 Patient II:4

**Figure 4:**
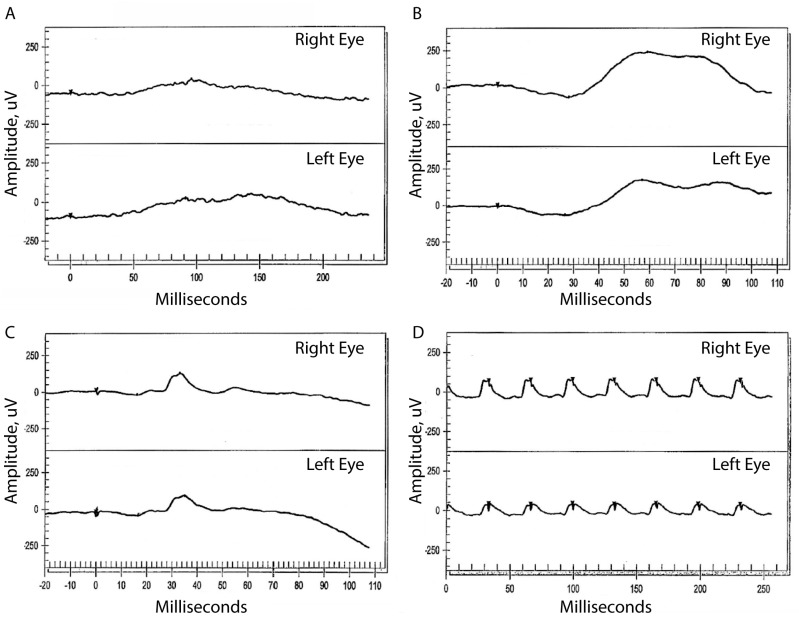
Electroretinogram (ERG). ERG responses from II:4 at age 47 show moderately reduced but measureable amplitudes in both eyes in response to (**A**) dim (−24 dB) scotopic flash, (**B**) mixed (0 db) scotopic flash, (**C**) single 0 dB photopic flash, and (**D**) 30 Hz 0 dB flicker stimuli.

The younger sister of II:2 was a 54-year-old woman who first presented at the age of 47 with decreased night vision for several years. She denied any hearing loss. Past ocular history was significant for longstanding preretinal fibrosis in the left macula. Past medical history was significant for diabetes mellitus type II controlled with oral medications, without significant diabetic retinopathy in either eye. BCVA was 20/25 in both eyes; intraocular pressures and anterior segment examination were normal in each eye. Kinetic visual fields showed mid-peripheral ring scotomas with preserved central islands (Figure 2C–D). Dilated fundus exam showed RPE change along the temporal arcades in each eye and a dense epiretinal membrane in the fovea along the superotemporal arcade with an overlying vitreous opacity in the left eye only (Figure 3E). Pigmentary changes were present along the inferotemporal arcade in the left eye. SD-OCT showed a preserved ONL, ELM, and inner segment/outer segment (IS/OS) junction band at the fovea with loss of the IS/OS junction band beginning within 10 degrees from the fovea in each eye (Figure 3G, arrows). ERG showed reduced but measurable amplitudes with rod-greater-than-cone dysfunction in each eye (Figure 4).

#### 3.2.3 Patient II:6

A 66-year-old female maternal second cousin of II:2 and II:4 first reported blurry vision at the age of 37. She denied any hearing loss or skeletal abnormalities. At age 39, BCVA was 20/40 in the right eye and 20/50 in the left eye. Dilated fundus exam showed a few vitreous cells and cystoid macular edema (CME) in both eyes. She was diagnosed with bilateral chronic posterior uveitis, but extensive workup did not reveal a cause for the uveitis. At age 56, she began complaining of progressive night vision loss and peripheral vision loss; BCVA was 20/70 in both eyes and visual fields showed mid-peripheral scotomas (Figure 2E). Dilated fundus exam revealed and bone spicule pigmentary changes in the mid-periphery. SD-OCT showed CME with large cysts involving the fovea and loss of the IS/OS junction band beginning within 10 degrees from the fovea in each eye (Figure 3H).

### 3.3. Genetic Analysis

WES and WGS of the three affected (II:2, II:4 and II:6) and two unaffected (I:6, II:3) family members were performed. WES did not identify potentially pathogenic variants shared by all three affected members. However, a previously reported homozygous nonsense mutation, c.226C>T (p.Arg76*), in *KIZ* was observed in the two affected sisters (II:2 and II:4) [18].

**Figure 5:**
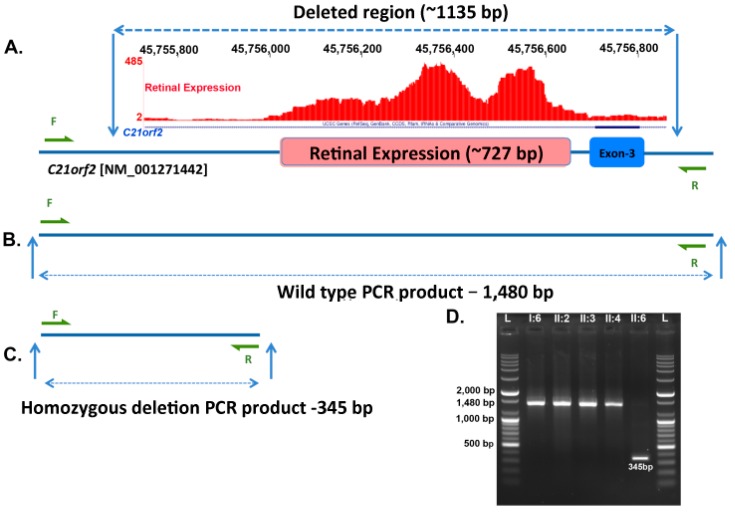
Segregation analysis of the deletion in *C21orf2*. (**A**) Region of *C21orf2* encompassing the 1,135 base pair (bp) homozygous deletion (Chr21: 45,755,728–45,756,862) observed in patient II:6 of the pedigree RF.L.11.10. A 727 bp region (Chr21: 45,755,983–45,756,710) within the 1,135 bp homozygous deletion is reported to encompass retina expressed sequence [17]. The retinal transcriptome ribonucleic acid-sequence (RNA-seq) coverage data is shown in red. In addition, this deletion also encompasses exon 3 of NM_001271442 transcript of *C21orf2*; (**B**,**C**) Region of *C21orf2* amplified by PCR to detect the presence of wild type or mutant alleles; (**D**) Gel electrophoresis of the PCR products of *C21orf2* deleted region in members of RF.L.10.11 family. Presence of the 345 bp PCR product in II:6 indicated the presence of the homozygous deletion in *C21orf2* whereas the other members had wild type alleles.

WGS identified about four million variants in each individual, but filtering these did not find potential disease-causing variants shared by all three affected members. Further analysis of structural variants revealed a novel 1.135 kb homozygous deletion (in hg19, Chr21: 45,755,728– 45,756,862) in *C21orf2* in the affected second cousin (II:6). A 727 bp region in this deletion (Chr21: 45755984-45756710) is reported to encode a transcript that is expressed in the retina [19] (Figure 5A and Figure S1). The remaining portion of the deletion encompasses exon 3 of *C21orf2* (NM_001271442), which is expressed at low levels in the retina (Figure 5A and Figure S1) [19]. The region spanning the deletion was amplified using primers flanking this region. This amplification reaction generates a 1480 bp product corresponding to the wild type allele (Figure 5B), and a 345 bp PCR product corresponding to the wild type allele with a 1.3 kb deletion (Figure 5C). The PCR products were analyzed by gel electrophoresis to detect the presence of wild type and mutant alleles (Figure 5D). The presence of wild type allele was observed in individuals I:6, II:2, II:3 and II:4, while II:6 showed the presence of a 345 bp PCR product, suggesting the presence of a homozygous deletion in *C21orf2*. Sequencing this product revealed the exact size of the deletion to be 1135 bp (data not shown).

**Figure 6:**
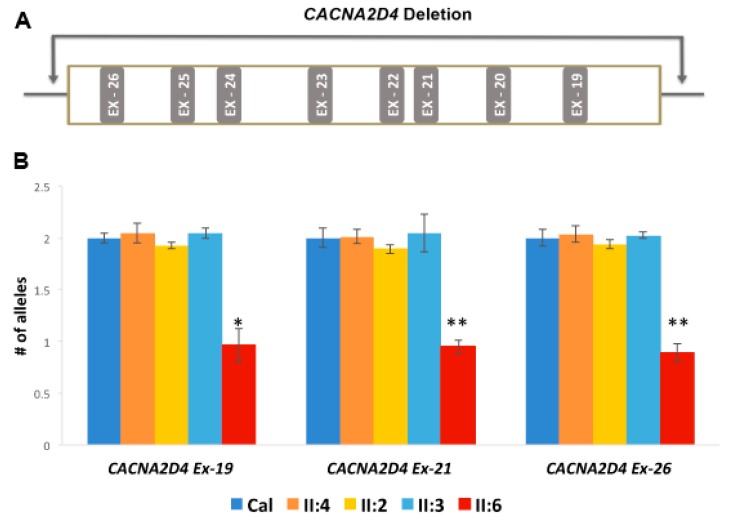
Quantitative polymerase chain reaction (qPCR) analysis of the *CACNA2D4* sequence with a large heterozygous deletion in pedigree RF.L.11.10. (**A**) Region spanning a 30 kb heterozygous deletion (Chr12: 1,949,399–1,980,050) observed in *CACNA2D4* gene in patient II:6 in pedigree RF.L11.10. This deletion includes 8 exons (exon 19 to exon 26) of the *CACNA2D4* gene; (**B**) qPCR of exons 19, 21 and 26 of *CACNA2D4* indicated the presence of two wild type alleles of *CACNA2D4* in II:2, II:3 and II:4; and a single copy of *CACNA2D4* exon 19, 21 and 26 in II:6. The qRT-PCR was normalized to two reference genes, *GPR15* and *ZNF80* and Control human genomic DNA was used as calibration control (Cal) to determine the copy number of each allele present in the test samples. * *p* < 0.05; ** *p* < 0.01.

A novel 30.651 kb heterozygous deletion (Chr12: 1949399–1980650-Hg19) that includes exons 19 to 26 of *CACNA2D4* was also identified in II:6 (Figure 6A). Quantitative PCR analysis of representative exons 19, 21 and 26 of *CACNA2D4* indicated that II:2 and II:4 had two copies, while II:6 had only one copy of these exons. These findings suggest the presence of a 30 kb deletion in II:6 in the heterozygous state (Figure 6B). This deletion also includes part of the potential regulatory region of the gene leucine rich repeats and transmembrane domains 2 (*LRTM2*). The role of this gene in RD is not known and additional analysis is needed to determine the impact of the chromosome 12 deletion on *LRTM2*.

In summary, the genetic analysis revealed that mutations in two independent genes underlie the pathology of RD in pedigree RF.L.10.11. II:2 and II:4 with the *KIZ* mutation did not carry the deletions in *C21orf2* or *CACNA2D4*, while II:6 with the *C21orf2* and *CACNA2D4* deletions did not carry the *KIZ* nonsense mutation.

## 4. Discussion

This study identified two independent homozygous mutations, in *KIZ* and *C21orf2*, associated with autosomal recessive RP in a non-consanguineous pedigree. The three affected family members all shared similar clinical characteristics, such as nyctalopia, mid-peripheral scotomas, and bone spicules, though the age of onset of symptoms and clinical presentation varied. WES and WGS revealed two separate, homozygous mutations segregating with disease in the three affected women. WES did not find potentially pathogenic variants shared by all three affected members, but WGS identified a homozygous deletion in *C21orf2* and an additional heterozygous deletion in *CACNA2D4*.

The homozygous nonsense mutation in *KIZ* (c.226C>T; p.Arg76*) was previously reported in two families with recessive rod-cone dystrophy: one family of North African Sephardic Jewish origin and the other of Spanish ancestry [18]. The current study reports this mutation in a family of Ashkenazi Jewish descent. Clinical findings in the two previously reported families were similar to the current study and typically seen in RP: nyctalopia, mid-peripheral visual field loss, and pigmentary changes in the peripheral retina. Although disease onset was earliest in II:2, both II:2 and II:4 shared early development of nyctalopia and progressive mid-peripheral visual field loss with central visual field constriction and ERG evidence of rod greater than cone dysfunction. At later stages of disease progression, II:2 and II:4 had central scotomas with preserved peripheral visual fields. Both II:2 and II:4 had epiretinal membranes, with dense preretinal fibrosis extending from the disc into the vitreous in II:4 (Figure 3E, right panel). II:2 and II:4 share features previously described in patients with *KIZ*-related RP, such as pigmentary changes in the peripheral retina and atrophic changes in the central macula with a perifoveal ring of increased autofluorescence (Figure 3B) and outer segment loss (Figure 3D,G) [18]. However, II:2 demonstrated preservation of the ONL and IS at the fovea despite visual acuity of 20/800 due to foveal OS loss (Figure 3D). Prior reports of *KIZ*-related RP also describe patients who developed nyctalopia in their late teens and undetectable ERG responses by age 35, while others retain visual acuity of 20/20–20/40 by age 50, decreased central retinal sensitivity with visual field constriction, preserved islands of perception peripherally and foveal outer retinal preservation [18]. Analysis of sequence variants in II:2 and II:4 identified 16 additional rare and potentially pathogenic variants (Table S2) that are discordant in zygosity in these siblings. The impact of these additional variants is unknown. If they are indeed pathogenic, these variants may contribute to variation in the phenotype. Additional studies are needed to determine their effect on the clinical phenotype.

The current study contributes new insight into the impact of *KIZ* mutations on foveal cones. The patient with the most advanced *KIZ*-related RD in the current study had central vision loss with preserved visual field in the far periphery associated with loss of photoreceptor outer segments in the macula. However, the ONL, ELM, and IS were preserved in macular SD-OCT scans through the fovea. This suggests that even in advanced disease, cone nuclei and inner segments may persist at the fovea, which may be amenable to restorative therapies [20].

*KIZ* encodes a centrosomal protein kizuna [21], and immunohistology revealed kizuna localizes to the basal body of cilia in human fibroblasts, suggesting it may play a role in photoreceptor connecting cilia [18]. Moderate hearing loss was described previously in one patient by age 50, and II:2 also had mild hearing loss in the lower and mid-frequency tones [18], suggesting *KIZ* may be important for auditory cilia in addition to photoreceptor cilia maintenance.

II:6 presented with severe CME beginning at age 39, but later also developed nyctalopia, mid-peripheral scotomas, and bone spicules (Table 1, Figure 2 and Figure 3). *C21orf2* mutations have been reported in patients with early onset autosomal recessive RP and posterior staphyloma from Saudi Arabia [22], and patients with cone rod dystrophy and RP from Japan [23]. CME was not reported previously and prior reports documented more severe retinal degeneration than was observed in the present study. *C21orf2* mutations have also been reported in patients with syndromic forms of retinal degeneration, including Jeune syndrome [24] and axial spondylometaphyseal dysplasia [25,26]. Homozygous mutations in the *CACNA2D4* gene have been associated with autosomal recessive cone rod dystrophy [27,28] and nonprogressive inner retinal cone system dysfunction [29], but carriers with only one mutation in the *CACNA2D4* gene had no symptoms [28,29]. The effect of a heterozygous deletion in *CACNA2D4* in a patient with homozygous, likely pathogenic deletions in the *C21orf2* gene is not known. The phenotype of patient II:6 is consistent with the phenotype reported in patients with *C21orf2*, indicating that the presence of *CACNA2D4* heterozygous deletion may not exert significant influence on the RD phenotype due to *C21orf2* mutations.

Similar to kizuna, *C21orf2* encodes a protein that localizes to photoreceptor-connecting cilia [22]. Although many ciliopathies affect organ systems, in addition to causing retinal degeneration [23,24,25], many affect the retina exclusively, such as in certain forms of RP or Leber congenital amaurosis [30]. All three patients in the current study had ocular manifestations including nyctalopia, mid-peripheral visual field loss, and retinal pigmentary changes, without extraocular findings suggestive of a syndromic disease, although II:2 developed mild hearing loss by age 52, and prior studies in *KIZ*–related RP reported moderate hearing loss in one patient by age 50 [18].

## 5. Conclusions

In summary, the homozygous nonsense mutation observed in the *KIZ* gene and the deletion in the *C21orf2* are likely to be sufficient to cause disease independently. The effect of the heterozygous *CACNA2D4* deletion is unknown.

This study underlies the importance of comprehensive genetic analysis. Common methodologies like Sanger sequencing, and in certain cases exome sequencing may not efficiently detect large structural changes in the genome such as the deletions observed in *C21orf2* and *CACNA2D4*. Particularly, the WES failed to detect the *C21orf2* deletion in this study, as exome capture probes used did not cover the majority of this region. In such cases, utilizing whole genome sequencing or comparative genomic hybridization analysis may be necessary to detect these mutations. Occurrence of multiple homozygous mutations in more than one gene in a single pedigree is not common. Employing next generation genetic analysis tools including the efficient exome capture probes or whole genome analysis and availability of information on transcripts expressed in tissue of interest may enable the discovery of these rare genotypes involving multiple genes in a family and improve molecular diagnosis. Additionally, patients should undergo thorough ophthalmic examination to characterize the phenotypic presentations of RD. As we gain a deeper understanding of the genetic causes of RD, the ability to correlate clinical findings with genotype may allow more targeted diagnostic testing and potential therapies in the future.

## Figures and Tables

**Table 1:** Clinical Characteristics.

Patient ID/Gender	Age (Years)	Age at First Sx (Years)	BCVA	Slit-Lamp Exam	Dilated Fundus Exam	Genetics
			OD, OS			
II:2/F	60	26	20/800 20/800	OU: mild NS cataract	OU: mild disc pallor, cellophane ERM, bone spicules, no CME	Gene: *KIZ*
						Chr20: 21136463
						c.226C>T (p.Arg76*)
II:4/F	54	44	20/25 20/25	OU: normal	OD: normal	Gene: *KIZ*
					OS: dense ERM with fibrosis	c.226C>T (p.Arg76*)
II:6/F	66	39	20/100 20/200	OU: normal	OU: bone spicules, CME	Gene: *C21orf2*
						Homozygous 1.135 kb deletion
						Chr21: 45,755,728–45,756,862
						Gene: *CACNA2D4*
						Heterozygous 30.651 kb deletion
						Chr12: 1,949,399–1,980,050

BCVA: best corrected visual acuity; CME: cystoid macular edema; ERM: epiretinal membrane; NS: nuclear sclerotic; OD: right eye; OS: left eye; OU: both eyes; Sx: symptoms.

## Supplementary Materials

The following are available online at www.mdpi.com/2073-4425/8/9/210/s1. Table S1, List of primers used to amplify *CACNA2D4* and *C21orf2* genes, A: List of primers used in quantitative polymerase chain reaction (qPCR); B: List of primers used to amplify the allele with *C21orf2* deletion. Table S2: List of rare and potentially pathogenic variants present in II:2 and II:4; AAChange: amino acid change; ALT: alternative allele; Chr: chromosome; N/A: not available; REF: reference allele; 1: Heterozygous; 2: Homozygous. Figure S1: Human retinal expression level in different transcripts of *C21orf2*.
