# Correction: Gustafson et al., Whole Genome Sequencing Revealed Mutations in Two Independent Genes as the Underlying Cause of Retinal Degeneration in an Ashkenazi Jewish Pedigree. *Genes* 2017, *8*, 210

**DOI:** 10.3390/genes8100286

**Published:** 2017-10-23

**Authors:** Kevin Gustafson, Jacque L. Duncan, Pooja Biswas, Angel Soto-Hermida, Hiroko Matsui, David Jakubosky, John Suk, Amalio Telenti, Kelly A. Frazer, Radha Ayyagari

**Affiliations:** 1 Ophthalmology, University of California, San Francisco, San Francisco, CA 94143-0730, USA; kevingustafson11@gmail.com; 2 REVA University, Bengaluru, Karnataka 560034, India; pobiswas@ucsd.edu; 3 Shiley Eye Institute, University of California, San Diego, La Jolla, CA 92093-0946, USA; ashermida@ucsd.edu (A.S.-H.); jjsuk@ucsd.edu (J.S.); rayyagari@ucsd.edu (R.A.); 4 Biomedical Sciences Graduate Program, University of California, San Diego, La Jolla, CA 92093, USA; hmatsui@ucsd.edu (H.M.); djakubos@ucsd.edu (D.J.); kafrazer@ucsd.edu (K.A.F.); 5 Human Longevity, Inc., San Diego, CA 92121, USA; atelenti@humanlongevity.com; 6 Department of Pediatrics, Rady Children’s Hospital, Division of Genome Information Sciences, San Diego, CA 92093, USA

Following publication of our article [1], we identified discrepancies between the pedigree shown in Figure 1 and the rest of the text. We modified the pedigree displayed in Figure 1 to respond to comments from the reviewers, but failed to update the figure legend, text, Figures 5 and 6 and supplementary Table S2 for consistency with the revised pedigree shown in Figure 1. These errors do not change the main findings and conclusions reported in our paper, but must be corrected for consistency of the pedigree with the rest of the data in the manuscript. The details of the errors are listed below:

(1) In the legend of Figure 1, we referred to the affected family members shown in the pedigree as II:1, II:3 and II:4, respectively, as II:2, II:4 and II:6. In addition, an unaffected family member who underwent genetic testing shown in the pedigree as II:2 was erroneously described as II:3. We have modified the legend to Figure 1, as well as the Materials and Methods description in Section 2, the Results in Section 3 including Table 1, figure legends for Figures 2, 3 and 4, and Figures 5 and 6 along with their legends, for consistency with the pedigree shown in revised Figure 1. Finally, we modified Supplementary Table S2 and Section 4, Discussion, to update the family members throughout the manuscript for consistency with the revised pedigree shown in Figure 1. The revised legend of Figure 1 is shown with the figure below.

(2) We deleted a paragraph that had been duplicated in Section 3.3.

The changes do not affect the scientific results. The manuscript will be updated and the original will remain online on the article webpage, with a reference to this Correction.

## Figures and Tables

**Figure 1:**
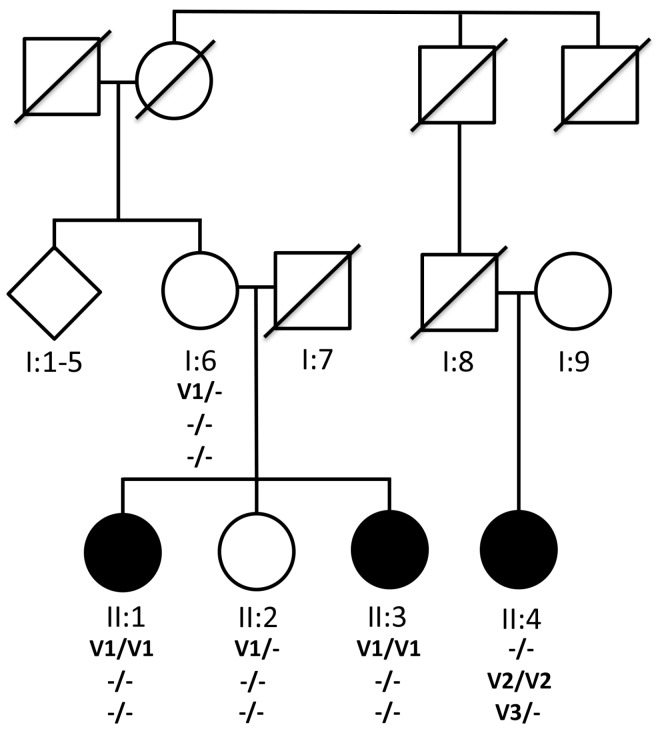
Pedigree RF.L.11.10 and segregation of mutations in *KIZ* and *C21orf2* with recessive RD. I:1–5 represents elder siblings (three unaffected males and two unaffected females) of I:6. (-) Indicates presence of wild type allele where as V1, V2 and V3 indicate the mutant alleles. The homozygous nonsense mutation p.Arg76* in *KIZ* (V1) segregated with disease in one branch of the family with affected members II:1 and II:3. A 1.1Kb homozygous deletion V2 (Chr21: 45,755,728–45,756,862) in *C21orf2* gene was observed in II:4 from a different branch of the pedigree RF.L.11.10. An additional 30Kb heterozygous deletion V3 (Chr12: 1,949,399–1,980,050) in *CACNA2D4* gene was also observed in the affected member II:4.

## References

[1] Gustafson K., Duncan J.L., Biswas P., Soto-Hermida A., Matsui H., Jakubosky D., Suk J., Telenti A., Frazer K.A., Ayyagari R. (2017). Whole Genome Sequencing Revealed Mutations in Two Independent Genes as the Underlying Cause of Retinal Degeneration in an Ashkenazi Jewish Pedigree. Genes.

