# Supplementary material for: Whole Genome Sequencing Revealed Mutations in Two Independent Genes as the Underlying Cause of Retinal Degeneration in an Ashkenazi Jewish Pedigree
Source: Genes (Basel). 2017 Aug 24;8(9):210. doi: 10.3390/genes8090210 (PMC5615344; doi:10.3390/genes8090210)
Supplement: Supplementary file 1 [file genes-08-00210-s001.zip › Table S1_Primers_New.docx]

Table S1: List of Primers used to amplify *CACNA2D4* and *C21orf2* genes;

A. List of Primers used in q-PCR:

| Gene | Forward primer (5’→3’) | Reverse primer (5’→3’) | Amplicon length (bp) |
| --- | --- | --- | --- |
| *CACNA2D4* Exon 26 | CCCATGACTGGGTTGTCTCT | ATGGCTGTCCTCTTGTCCAC | 103 |
| *CACNA2D4* Exon 21 | CCCTGAATGTGGACTCTGGT | CCTGTCTCCCTCCCATTTTT | 105 |
| *CACNA2D4* Exon 19 | AAGCGAGTTCTTTTCCTGACC | CTCACCCCGTGCAAATAAAG | 96 |
| *ZNF80* | CTGTGACCTGCAGCTCATCCT | TAAGTTCTCTGACGTTGACTGATGTG | 120 |
| *GPR15* | GGTCCCTGGTGGCCTTAATT | TTGCTGGTAATGGGCACACA | 101 |

B. List of Primers used to amplify the allele with *C21orf2* deletion:

| Gene | Forward primer (5’→3’) | Reverse primer (5’→3’) | Amplicon length (bp) |
| --- | --- | --- | --- |
| *C21orf2* | TACACAGGGATCGCATACCA | TGGAGGTTTCCAGGTTATGTC | 1480 |
