# Supplementary material for: Whole Genome Sequencing Revealed Mutations in Two Independent Genes as the Underlying Cause of Retinal Degeneration in an Ashkenazi Jewish Pedigree
Source: Genes (Basel). 2017 Aug 24;8(9):210. doi: 10.3390/genes8090210 (PMC5615344; doi:10.3390/genes8090210)
Supplement: Supplementary file 1 [file genes-08-00210-s001.zip › Table S2_List of Variants_New.docx]

Table S2: List of rare and potentially pathogenic variants present in II:2 and II:4.

| **Gene** | **Chr** | **Position** | **RsID** | **REF** | **ALT** | **ExAC** | **AAChange** | **II:2** | **II:4** |
| --- | --- | --- | --- | --- | --- | --- | --- | --- | --- |
| *AXDND1* | chr1 | 179335262 | N/A | GTA | G | N/A | - | 1 | 2 |
| *UNC80* | chr2 | 210707088 | rs199664663 | T | G | 0.001327 | p.Ser1126Arg | 1 | 2 |
| *DCHS2* | chr4 | 155244401 | rs199840326 | TTTTG | T | N/A | p.Asn1365fs | 1 | 2 |
| *OR13C2* | chr9 | 107367392 | rs143198170 | TGTTA | T | N/A | p.Asn171fs | 1 | 2 |
| *GPRIN2* | chr10 | 46999591 | rs112620425 | C | CATGAGGGAG | N/A | p.Met238_Glu240dup | 2 | 1 |
| *PTGES3* | chr12 | 57080664 | rs374710214 | CAT | C | N/A | p.Met1fs | 1 | 2 |
| *ASCL1\|PAH* | chr12 | 103352171 | rs369257660 | C | CGCA | N/A | p.Gln62dup | 2 | 1 |
| *DDHD1* | chr14 | 53619480 | rs140904345 | T | TGCCGCC | N/A | p.Gly111_Gly112dup | 1 | 2 |
| *RAI1* | chr17 | 17697101 | N/A | AG | A | N/A | p.Gln280fs | 2 | 1 |
| *REXO1* | chr19 | 1827020 | N/A | T | TGGA | N/A | p.Ser589dup | 1 | 2 |
| *MUC16* | chr19 | 9028340 | rs199525769 | T | A | 0.0007102 | p.Asp12151Val | 1 | 2 |
| *ZNF527* | chr19 | 37879852 | rs376931538 | C | CTGTG | N/A | p.Pro301fs | 1 | 2 |
| *HRC* | chr19 | 49657710 | rs61355957 | ACAT | A | N/A | p.Asp261del | 1 | 2 |
| *HRC* | chr19 | 49657889 | rs147238387 | T | TTCC | N/A | p.Glu202dup | 1 | 2 |
| *SSC5D* | chr19 | 56029616 | rs150781976 | C | CCCA | N/A | p.Thr1326dup | 1 | 2 |
| *CTSA* | chr20 | 44520237 | N/A | CCTG | C | N/A | p.Leu37del | 1 | 2 |

Abbreviations: AAChange - Amino acid Change; ALT - Alternative Allele; Chr - Chromosome; N/A - Not Avaiavle; REF - Reference Allele.

1: Heterozygous; 2:Homozygous.
