# Supplementary figures and images for: Whole Genome Sequencing Revealed Mutations in Two Independent Genes as the Underlying Cause of Retinal Degeneration in an Ashkenazi Jewish Pedigree
Source: Genes (Basel). 2017 Aug 24;8(9):210. doi: 10.3390/genes8090210 (PMC5615344; doi:10.3390/genes8090210)

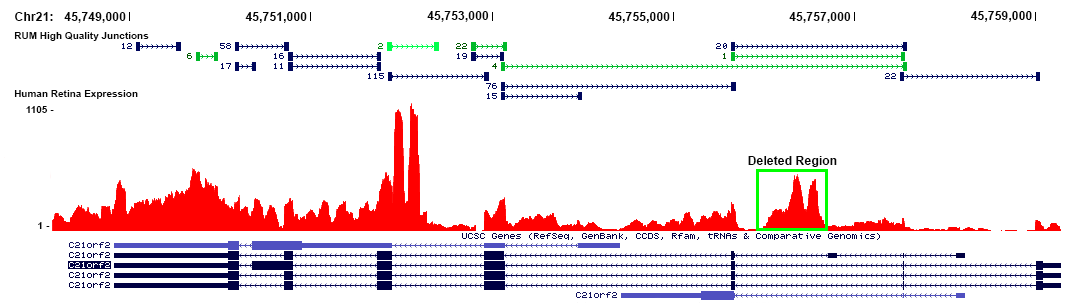

Supplement: Supplementary file 1 [file genes-08-00210-s001.zip › Figure S1_New.tif]
